# Mitochondrial Medicine and the Neurodegenerative Mitochondriopathies

**DOI:** 10.3390/ph2030150

**Published:** 2009-12-03

**Authors:** Russell H. Swerdlow

**Affiliations:** Departments of Neurology and Molecular and Integrative Physiology, University of Kansas School of Medicine, Kansas City, MO, Kansas 66160, USA; Email: rswerdlow@kumc.edu; Tel.: +1-913-588-0685; Fax: +1-913-588-0681.

**Keywords:** mitochondria, mitochondrial biogenesis, mitochondriopathies, neuro-degenerative diseases, oxidative stress

## Abstract

Neurodegenerative diseases are a common late-life scourge for which disease-modifying treatments are sorely needed. Mitochondrial perturbation is commonly observed in these diseases, so pursuing treatment development strategies that target mitochondria or processes affected by mitochondria seems reasonable. This review discusses the rationale underlying past and current efforts to treat neurodegenerative diseases using mitochondrial medicine, and tries to predict how future efforts might proceed.

## 1. Neurodegenerative Diseases Are Mitochondriopathies

### 1.1. Brief overview of the neurodegenerative diseases

Neurodegenerative diseases are characterized by the progressive loss of neuron populations. These disorders afflict all age groups. There are rare childhood neurodegenerations, neurodegenerations that present in early or mid-adulthood, and others that manifest in late-life. The latter neurodegenerations are particularly common. A case can be made that among the oldest old it is more common to have a neurodegenerative disease than it is to not have one [1]. 

Individual neurodegenerative diseases are defined on both clinical and pathological levels. Diagnoses are most reliable when a specific, recognizable clinical syndrome occurs in conjunction with specific brain histopathology. Diagnostic limitations arise from the fact that substantial clinical overlap exists between some disorders, clinical presentations are occasionally atypical or simply hard to characterize, and various pathologic features may co-exist in the brains of affected patients. The neurodegenerative disorder dementia with Lewy bodies (DLB) is a straightforward example of this. Dementia is a central feature of DLB, and parkinsonism is a frequently cited core feature [2]. Patients diagnosed with DLB often fulfill clinical criteria for both Alzheimer’s disease (AD) and Parkinson’s disease (PD). Their brains also show neuritic plaques and neurofibrillary tangles, findings typically associated with AD, as well as Lewy bodies, which are typically associated with PD. 

In addition to clinical and pathology overlap, molecular overlap also occurs. One recurrent theme is protein aggregation. Protein aggregation can occur extracellularly, in the cytosol, in the nucleus, or in combination. Amyloid plaques are examples of extracellular protein aggregation. In AD, plaques contain the beta amyloid (Aβ) protein, which is itself produced through the enzymatic digestion of the larger amyloid precursor protein (APP). Diseases with amyloid aggregation qualify as “amyloidoses”. 

Tau, tar binding protein 43 (TDP43), and synuclein form cytosolic aggregations. Tau comprises the “tangles” seen in multiple neurodegenerative diseases including AD, frontotemporal dementia (FTD), progressive supranuclear palsy (PSP), and corticobasal degeneration (CBD). TDP43 aggregation occurs in FTD and amyotrophic lateral sclerosis (ALS). Synuclein, the primary constituent of Lewy bodies, aggregates in PD, DLB, and muti-system atrophy (MSA). Synuclein is also found extracellularly in AD plaques. These disorders may be variably classified as “tauopathies”, “TDP43-opathies”, and “synucleinopathies”. Nuclear aggregations can occur in neurodegenerative diseases caused by triple repeat expansions. For example, in Hungtington’s disease (HD), huntingtin protein (htt) aggregates in neuron nuclei.

Another recurrent molecular theme in neurodegenerative diseases is mitochondrial perturbation. Observed mitochondrial perturbations are believed to associate with mitochondrial dysfunction. The role mitochondrial perturbation plays in neurodegenerative diseases is sometimes unclear and may differ from disease to disease. In some disorders it appears to initiate neuronal dysfunction and ultimately degeneration. In others it possibly plays an intermediary but unique contributory role. Occasionally, it may represent a generic but still important consequence of more upstream cascades. The term “neurodegenerative mitochondriopathies” was previously proposed to categorize neurodegenerative diseases that feature mitochondrial perturbation [3,4]. The following section provides a brief overview of mitochondrial defects seen in various neurodegenerative diseases. 

### 1.2. Mitochondrial perturbation occurs in multiple neurodegenerative diseases

In general, mitochondrial perturbation can arise in several ways. Mutations in mitochondrial DNA (mtDNA) genes, mutations in nuclear genes encoding mitochondrial proteins, and defects in intergenomic signaling can all interfere with mitochondrial function. Gain of function mutations in proteins that normally have no or limited mitochondrial interaction may cause them to directly or indirectly hinder mitochondrial function. Loss of function mutations may deprive mitochondria of necessary support. Changes in the cytosolic milieu may also disrupt mitochondria. Functional defects may involve transport of proteins and substrates into and out of the mitochondria, or else substrate utilization. Mitochondrial perturbations may be fairly non-specific or specific. Examples of non-specific changes include mitochondrial depolarization, enlargement, and ultrastructure changes. Examples of specific defects include diseases in which one but not other respiratory enzymes have altered function. 

Both specific and non-specific mitochondrial perturbations are observed in AD [5]. The most specific defect involves a reduction in the complex IV (cytochrome oxidase) Vmax activity [6]. This defect is seen in brains and non-degenerating tissues of AD patients. Most studies suggest Vmax activities of other electron transport chain (ETC) enzymes are normal. When mitochondria from AD or control subject platelets are transferred to mtDNA-depleted, respiratory-incompetent cells to form cytoplasmic hybrid (cybrid) cell lines, respiratory function is restored but the cytochrome oxidase Vmax activity in cybrid lines containing AD subject mtDNA is less than it is in cyrid lines containing control subject mtDNA [7,8,9]. This suggests the AD cytochrome oxidase defect arises from mtDNA, although the exact AD mtDNA problem is unclear. Other forms of AD mitochondrial dysfunction are documented, including reductions in the pyruvate dehydrogenase complex Vmax and the ketoglutarate dehydrogenase Vmax [5]. Oxidative stress is increased in AD subject tissues, and may reflect mitochondrial dysfunction. AD brains also show altered mitochdondrial ultrastructure, increased levels of the 5 KB “common” mtDNA deletion, reduced numbers of intact mitochondria, increased numbers of degrading mitochondria, and altered mitochondrial fission-fusion dynamics [10,11].

From a cause-and-effect perspective AD is particularly perplexing because more than one type of AD may exist [1]. Rare autosomal dominant cases that arise from APP mutations can be modeled in genetically altered mice via overexpression of a mutated human APP transgene (tg2576 mice). Mitochondrial physiology is altered in these mice. One early change indicates mitochondrial proliferation, which suggests expression of the mutant transgene negatively impacts mitochondrial performance and induces a compensatory response [12]. Similar results were more recently reported from triple transgenic mice that also express mutant presenilin 1 and tau transgenes [13]. Other studies indicate in tg2576 mice Aβ interferes with the mitochondrial permeability transition as well as the function of the mitochondrial abeta-binding alcohol dehydrogenase (ABAD) enzyme [14,15]. In humans, APP is found in the mitochondrial membrane and Aβ is found inside the mitochondria [16,17,18]. However, it has also been shown that mitochondrial dysfunction can increase Aβ production [19,20]. Some propose that in sporadic, late onset AD upstream mitochondrial dysfunction may account for a downstream amyloidosis [21,22].

PD also is characterized by specific and non-specific mitochondrial perturbations. Complex I activity is consistently found to be reduced both in the brains and peripheral tissues of PD patients, while activities of other ETC enzymes are generally not [4,23,24]. The PD complex I defect is believed to be particularly relevant since complex I inhibition produces parkinsonism and dopaminergic neurons appear particularly vulnerable to complex I inhibition [25,26]. Similar to what is seen in AD, when mitochondria from PD subject platelets are used to produce cybrid cell lines, respiratory function is restored but the complex I Vmax activity is less than in cybrid lines containing control subject mtDNA [7,27,28,29]. This suggests the PD complex I defect arises from mtDNA. Although the exact PD mtDNA problem is unclear, PD subject substantia nigra neurons have increased levels of mtDNA deletions and their brains show microheteroplasmic mutations in specific stretches of the mtDNA ND5 gene [30,31,32,33]. Oxidative stress markers are elevated in PD subject tissues, and cybrid studies further suggest this arises as a consequence of complex I dysfunction [7,27,34]. 

PD, like AD, is etiologically heterogeneous with at least one common sporadic form and a number of rare Mendelian forms. Mutations that cause several of the Mendelian forms affect proteins that either localize to mitochondria or otherwise appear to influence mitochondrial function [35].

Other sporadic neurodegenerative diseases are associated with specific ETC Vmax reductions. Amyotrophic lateral sclerosis is most generally associated with complex I dysfunction, although reduced cytochrome oxidase Vmax activities are occasionally reported [36,37]. PSP cybrids have a complex I Vmax defect, which suggests PSP subjects should themselves have reduced complex I activity [38,39]. Oxidative stress is increased in both these diseases. Mitochondrial dysfunction is also implicated in numerous Mendelian neurodegenerations. Multiple ETC Vmax activities are reportedly reduced in HD [40]. It is unclear whether htt mutation disrupts mitochondrial function by directly interacting with the organelles themselves, or indirectly by affecting other proteins or genes that impact mitochondrial function [41,42]. Frataxin, the mutated gene that causes Friedreich’s ataxia (FA), encodes a protein that plays a role in mitochondrial iron homeostasis [43,44]. 

Neurodegeneration occurs in various mitochondrial encephalomyopathy disorders that are caused by specific mtDNA mutations [4]. The mitochondrial encephalomyopathies tend to occur in childhood or early adulthood. One of the best characterized mtDNA diseases is Leber’s hereditary optic neuropathy (LHON), a neuroanatomically discrete neurodegenerative disease of the optic nerve. 

The list of neurodegenerative mitochondriopathies is long and these disorders are more comprehensively described elsewhere [4]. Even from this brief overview, though, it is clear that mitochondrial perturbation is a common theme in neurodegenerative diseases. For disorders that qualify as neurodegenerative mitochondriopathies, pursuing treatment development strategies that target mitochondria or processes affected by mitochondria seems reasonable [3].

## 2. Mitochondrial Medicine

### 2.1. Definition and overview of mitochondrial medicine targets

“Mitochondrial medicine” describes efforts to directly manage mitochondrial dysfunction as well as attempts to directly or indirectly manage its consequences [45]. Early on, the well-recognized role of mitochondria in energy production gave rise to strategies designed to enhance electron transfer through the ETC. More recently, extensive mitochondrial integration within programmed cell death programs was demonstrated [46]. Attempts to abort programmed cell death cascades by blocking this integration are in vogue. Finally, oxidative stress is seen in many neurodegenerative diseases. Since oxidative stress may either arise as a consequence of mitochondrial dysfunction or else interfere with mitochondrial function, reducing oxidative stress qualifies as a form of mitochondrial medicine. 

**Figure 1:**
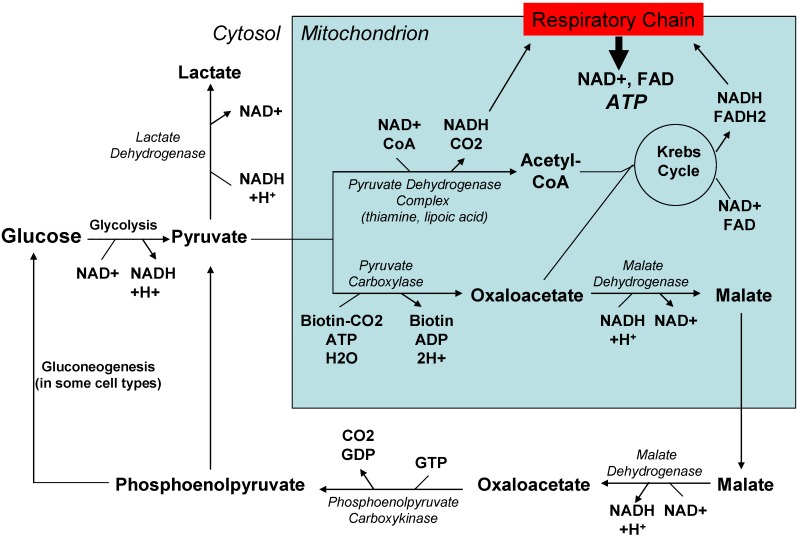
An integrated view of pathways involved in cell energy production.

Figure 1 provides an overview of pathways featured in energy production. Attempts to directly manage mitochondrial dysfunction by increasing energy production have traditionally included B vitamin supplements, since some B vitamins (riboflavin, or B2; niacin, or B3) are used to generate molecules that donate electrons to the mitochondrial respiratory chain. Other B vitamins (thiamine, or B1) are cofactors in biochemical reactions that generate bioenergetic pathway intermediates such as acetyl CoA. Thiamine, lipoic acid, and dichloroacetate have been used to enhance pyruvate dehydrogenase activity and increase acetyl CoA levels. Molecules capable of accepting electrons from or donating electrons to the respiratory chain (coenzyme Q, idebenone, vitamin K derivatives, methylene blue) have been used with the intent of augmenting reduced electron transport through the respiratory chain. 

Other approaches for enhancing cell energy supplies have been considered. Using acetyl donors like acetyl carnitine to increase Krebs cycle carbon flux is one approach [47,48]. Using the ketone body betahydroxybutyrate to encourage carbon entry into the Krebs cycle is another [49]. More indirect ways of buttressing cell energy reserves include administering compounds such as creatine that can store high energy phosphate bonds [50]. 

Neurodegenerative cell demise may occur via programmed cell death pathways [46]. These pathways are activated when mitochondria release proteins normally sequestered within them. Examples include cytochrome c, apoptosis initiating factor (AIF), and SMAC/DIABLO [51]. Release reportedly is mediated by or at least associated with the formation of an inner and outer mitochondrial membrane pore. To accomplish this “mitochondrial permeability transition” (MPT), the voltage dependent anion channel (VDAC; also called porin) protein of the outer mitochondrial membrane associates with the adenine nucleotide transporter (ANT) protein of the inner mitochondrial membrane [52,53]. This process depends on cyclophilin D, an intermembrane space protein that is also a requisite MPT component. Agents that can block the MPT or interfere with formation of the MPT pore, such as cyclosporine and minocycline, have been considered for the treatment of neurodegenerative diseases. 

Because defective mitochondria produce elevated amounts of reactive oxygen species (ROS), which causes oxidative stress, antioxidants have also been advocated for the treatment of mitochondrial dysfunction. Drugs used for their antioxidant potential include not only vitamin C, vitamin E, and β-carotene but also several of the electron acceptors already mentioned such as coenzyme Q and idebenone. 

### 2.2. Past and current experience

ETC enhancement was initially attempted in encephalomyopathy patients [3,54]. Reported outcomes vary. Case reports and case series typically report some degree of success. While the low prevalence of these disorders makes it difficult to conduct definitive controlled trials, in general the controlled trials that have been done failed to show clinical benefits. 

Using creatine to facilitate energy storage has been or is being tried in ALS, HD, and PD [3]. ALS subjects did not show demonstrable benefits. Although some trials of creatine in HD have suggested a small degree of efficacy, most have not [50]. Clinical studies of creatine in HD are still ongoing but a recent review of all available data concluded it probably does not help in HD [55]. A large study of creatinine in PD is currently underway. 

Mitochondrial medicine for the treatment of AD was advocated even before the AD cytochrome oxidase defect was discovered. In 1989, Swerdlow *et al*. reported AD brains can metabolize β-hydroxybutyrate and proposed using ketone bodies to treat AD [56]. At that time it was known AD brains had reduced glucose consumption. Because β-hydroxybutyrate negotiates the blood-brain barrier, accesses neuron mitochondria, produces acetyl CoA, and supplies acetyl groups to the Krebs cycle Swerdlow *et al*. concluded β-hydroxybutyrate could potentially provide an alternative to and mitigate the consequences of reduced AD brain glucose consumption [56]. Two decades later, the Food and Drug Administration ruled that the compound AC-1202 (ketasyn) could be marketed as a medical food for the treatment of AD. Ketasyn, a medium chain triglyceride, is converted by the liver to β-hydroxybutyrate and increases serum β-hydroxybutyrate levels in the absence of other dietary manipulations such as fasting or carbohydrate restriction. A double-blind, placebo-controlled trial of ketasyn in AD subjects was reported in 2009 [57]. Subjects underwent cognitive testing 45 and 90 days after starting in the study. APOE4-negative subjects receiving ketasyn performed better than those receiving placebo; for APOE4 carriers day 45 and 90 cognitive performances were comparable. For several reasons it is difficult to draw firm conclusions from this trial, but in general the possibility that some AD subjects did benefit from ketasyn cannot be excluded.

Coenzyme Q has been considered for neurodegenerative disease treatment based on its known participation in ETC function and its ability to act as antioxidant. An initial phase II trial of coenzyme Q in PD reported slowed progression, but no further supportive data have emerged since this trial [58]. Idebonone, a water soluble coenzyme Q analog, benefited cardiac hypertrophy in FA but had no impact on its neurologic signs and symptoms [59,60]. A small pilot study suggested coenzyme Q might potentially benefit PSP patients to a slight degree [61]. 

Antioxidants have been evaluated in retrospective epidemiologic studies, prospective epidemiologic studies, and actual clinical trials. Some epidemiologic observational studies suggest high dietary antioxidant intake reduces AD risk, but of course such studies do not allow definitive cause and effect inferences and are thus prone to confounding [62,63]. Studies associating AD risk with amounts of antioxidant supplement use have not shown a consistent benefit [64]. The most influential antioxidant trial to date is a study of high dose vitamin E on AD progression rates. After manipulating the data to account for baseline inequities in the treatment and placebo groups, it was concluded decline in the 2,000 IU per day vitamin E group was slower than it was in the placebo group [65]. Based mostly on these data, in 2001 the American Academy of Neurology released a dementia management practice parameter paper stating it was moderately convinced vitamin E could “delay the time to clinical worsening” and that 2,000 IU per day of vitamin E “should be considered in an attempt to slow progression of AD” [66]. However, a subsequent meta-analysis of vitamin E clinical trials suggested doses in excess of 400 IU per day could negatively impact overall health [67]. Based on this meta-analysis and the fact that any potential benefit of high dose vitamin E in AD is at best difficult to detect, enthusiasm for this treatment option has waned. More recently it was reported antioxidant supplements may reduce some of the benefits of physical fitness [68], further emphasizing the point that sustained high-dose antioxidant intake may adversely affect health [69]. To sum up existing data on the use of antioxidants in neurodegenerative diseases, some minimal or potential minimal successes are reported but in no case has antioxidant therapy proved transformative. 

Minocycline was shown to extend survival in a transgenic mouse model of autosomal dominant ALS (the G93A mouse that expresses a mutant human SOD1 transgene), and this benefit was believed to be mediated by effects on the MPT [70]. Although minocycline benefited the mice, in a placebo controlled study conducted on human sporadic ALS subjects those receiving minocycline actually did worse than those who received the placebo [71]. 

## 3. Ongoing and Future Efforts To Develop Neurodegenerative Disease Mitochondrial Medicine

### 3.1. New drugs for existing targets

Mitochondrial medicine has identified several pharmacologic targets, but manipulating these targets so far has not provided meaningful neurodegenerative disease-modifying treatments. This does not mean the targets are themselves flawed. As with any drug development effort, the form a drug is in when it reaches its target, the amount of drug that reaches the target, whether the drug even reaches its target, how effectively the drug affects the target, and what collateral effects the drug has all require consideration. 

As ongoing research advances what is known about a particular target, the approaches used to modify that target also evolve. Antioxidant-based therapeutic development provides a good example of this. There is emerging consensus that free radicals are not uniformly harmful. Numerous variants are recognized, compartmentalization is an issue, and it is clear radicals themselves play important physiologic roles [72,73]. It has accordingly been suggested if neurodegenerative disease oxidative stress is a consequence of mitochondrial dysfunction, it makes sense to direct therapeutic antioxidants to the mitochondrial source [74,75]. This can be accomplished by attaching antioxidants to cationic molecules; doing so allows the antioxidants to concentrate within negatively charged mitochondrial matrices. The most studied mitochondria-targeted antioxidant is MitoQ, which consists of coenzyme Q conjugated to a triphenyl alkyl phosphonium cation [76]. Human phase 2 clinical trials of MitoQ for PD and FA were announced several years ago but results, if any, have not been widely disseminated.

On the other end of the spectrum, drugs not developed as mitochondrial drugs have been recast as such. Dimebon, a drug originally developed as an antihistamine, is a case in point. Early phase clinical evaluations, including a phase 2 human trial, suggest dimebon may benefit AD patients [77]. If correct, it will be necessary to identify the responsible mechanism. Since dimebon has been shown to modify the MPT, a possible mitochondrial mechanism of action has been postulated [78]. It will be interesting to see how dimebon performs in definitive phase 3 testing. Additional studies of how dimebon affects mitochondrial function are underway. 

There is of course an alternative explanation to why mitochondrial medicine thus far has not provided effective neurodegenerative mitochondriopathy treatments. It is necessary to consider the possibility that most development efforts targeted phenomena that are perhaps at best downstream of and at worst compensatory adaptations (such as mitochondrial ROS production) to upstream mitochondrial failure or stress. If correct, then new mitochondrial medicine targets are needed. 

### 3.2. Enhancing aerobic metabolism

As far as developing treatments for the neurodegenerative mitochondriopathies goes, this reviewer finds the idea of increasing cell aerobic/anaerobic metabolism ratios particularly intriguing. Cell respiratory capacity declines with age [79], and this decline is exaggerated in neurodegenerative mitochondriopathies [3]. Two non-pharmacologic approaches, exercise and dietary restriction (DR), enhance aerobic infrastructure and have recognized health benefits [80,81,82,83,84,85,86,87]. While these interventions may not uniformly increase oxygen consumption, in at least some instances they do elevate aerobic set points. Delineating mechanisms through which exercise and DR produce their effects could provide a blueprint for how to accomplish this pharmacologically. In general, it appears aerobic metabolism can be enhanced in two ways. A cell can be induced to produce more mitochondria, or its existing mitochondria can be forced to respire more. 

#### 3.2.1. With mitochondrial biogenesis

DR, which promotes mitochondrial biogenesis, extends longevity in invertebrate and vertebrate species [88]. There is an apparent relation between life extension and cell respiratory status, as DR can activate the energy supply-sensing enzyme AMP kinase (AMPK) and consistently activates the redox state-sensing deacetylase enzyme SIRT1 [89,90]. These enzymes are suspected to play a role and perhaps even a key role in lifespan extension [91]. Free radicals are most likely also important. One study in *C. elegans* found antioxidants prevented 2-deoxyglucose, a glycolysis inhibitor that creates a DR-like state, from enhancing longevity [92]. 

Prolonged exercise also promotes muscle mitochondrial biogenesis. This associates with and probably mediates the endurance benefit that accompanies aerobic training [93]. Aerobic exercise also increases muscle free radical levels, and for this reason some athletes incorporate antioxidant vitamin supplements into their training regimen [94]. As is the case for antioxidants and DR, though, antioxidants seem to adversely affect at least some exercise-associated health benefits [68]. Data further suggest antioxidant supplements may mitigate the mitochondrial biogenesis that typically accompanies fitness training, thereby minimizing the ability of exercise to enhance physical endurance [95]. 

This review has so far considered antioxidants from the perspective of vitamin supplements such as vitamin E. Antioxidant enzyme systems also impact both cell and organelle free radical levels. Antioxidant enzyme expression is initiated by the same transcription programs that drive mitochondrial biogenesis [96]. In this respect mitochondrial biogenesis and oxidative stress create a feedback loop. If a cell requires more mitochondrial respiration to maintain energy homeostasis the existing mitochondria respire more, mitochondrial superoxide production increases, and oxidative stress signals the nucleus to increase both mitochondrial mass and antioxidant enzyme capacity. This likely explains why exercising muscles show increased free radical production but not increased oxidative damage [97]. It is further important to stress that although some chemicals are billed as antioxidants because they reduce free radical levels, their actual effects on respiratory function may be quite complex.

Resveratrol, a polyphenol compound, provides a good example of this. It is often referred to as an antioxidant, but its ability to reduce oxidative stress may arise indirectly through activation of SIRT1 and, at least in some tissues, mitochondrial biogenesis [98,99,100]. The use of polyphenols for the treatment of neurodegenerative mitochondriopathies is actively being explored. 

The thiazolidinedione drugs originally developed to treat type II diabetes have been considered for the treatment of neurodegenerative diseases. The thiazolidinediones, which include rosiglitazone and pioglitazone, are peroxisome proliferator activated receptor γ (PPARG) agonists. They do, however, also affect mitochondrial function and were shown in both *in vitro* and *in vivo* settings to enhance mitochondrial biogenesis [101,102,103]. A phase 2 trial of rosiglitazone in AD suggested APOE4-negative subjects might have benefited [104], but a phase 3 trial did not bear this out and efforts to develop thiazolidinediones as AD treatments have apparently been abandoned. Thiazolidinediones, though, poorly penetrate the blood barrier and it is uncertain whether the brain can achieve concentrations that optimally induce mitochondrial biogenesis [102].

#### 3.2.2. Without mitochondrial biogenesis

**Figure 2:**
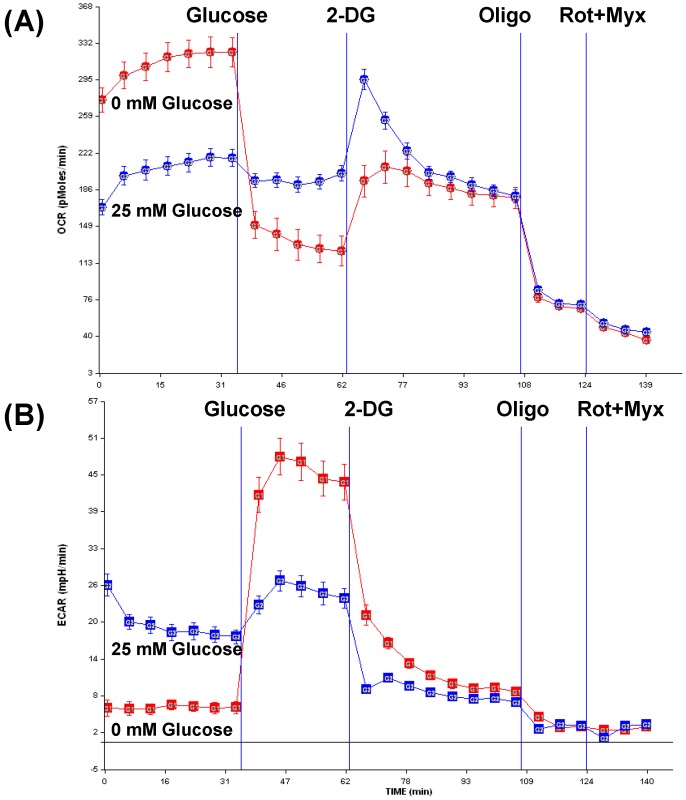
In human neuroblastoma cells, glucose starvation increases mitochondrial respiration without inducing obvious mitochondrial biogenesis. The data in this figure were produced using an XF24 Flux Analzyer (Seahorse Biosciences). At the start of the experiment SH-SY5Y cells were maintained in either normal DMEM medium (control cells) or in DMEM containing no glucose (starved cells); at the time these measurements were taken the starved cell had been without glucose for 15 hours. Glucose starved cell metabolic physiology is indicated by orange lines, and control cell metabolic physiology is indicated by blue lines. Panel (A) shows the oxygen consumption rates (OCR) of cells maintained under the two conditions, and panel (B) shows the extracellular acidification rate (ECAR) that is mostly due to lactate excretion. After baseline OCR and ECAR values are established, glucose was injected in the mediums, and this had a profound effect on the starved but not the control cells. The glycolysis inhibitor 2-deoxyglucose was next injected, which rapidly increased the control cell OCR to the level it was in the 15 hour starved cells. This was followed by injection of the ATP synthase inhibitor oligomycin, which terminates coupled mitochondrial respiration. Lastly, the complex I and III inhibitors rotenone and myxothiazol were used to stop the residual uncoupled mitochondrial respiration, so only non-mitochondrial oxygen consumption remains. After oligomycin and again after rotenone and myxothiazol the OCR values for both the control and starved cells were similar, indicating in the experiment starved and control cell numbers were equivalent. Additional details pertaining to this experiment are provided in the text. 2 DG=2 deoxyglucose; oligo=oligomycin; rot=rotenone; myx=myxothiazol.

Enhancing aerobic metabolism without inducing mitochondrial biogenesis may also provide a viable treatment option for the neurodegenerative mitochondriopathies. For example, in tumor cell lines it is certainly possible to increase cell respiration without inducing mitochondrial biogenesis. When human neuroblastoma SH-SY5Y cells are maintained under glucose deprivation conditions mitochondrial respiration increases until the maximum respiratory capacity is reached. Figure 2 shows the oxygen consumption rate (OCR) of SH-SY5Y cells maintained in medium containing 25 mM glucose and medium containing no glucose for 15 hours. Oxygen consumption is greatly increased in the glucose deprived cells. During this time lactate production in the glucose starved cells is, not surprisingly, virtually undetectable. The cells remain viable during this extended period, and presumably divert glutamine contained in the medium to the Krebs cycle and the production of ETC reducing equivalents.

When glucose is injected into the mediums, the starved cells rapidly reduce their OCR below that of the control cells, and increase lactate production above that of the control cells. This indicates the starvation intervention sensitized the cells to glucose consumption. When the glycolysis inhibitor 2-deoxyglucose is next added, the control cells rapidly increase their OCR and decrease their lactate production. With 2-deoxyglucose the control cell OCR actually approximates that of the 15 hour glucose-starved cells, suggesting that although the starved cell OCR was elevated for 15 hours no or little mitochondrial biogenesis occurred.

This experiment provides a cell culture simulation of animal DR. The failure to induce mitochondrial biogenesis in this case is potentially a consequence of the Warburg Effect that applies to tumor cells. The Warburg Effect refers to the tendency of tumor cells to generate their energy anaerobically through glycolysis and lactate formation, and at the expense of oxidative phosphorylation [105,106,107]. This metabolic pattern persists even when adequate oxygen is present. While the results described above may only apply to tumor cells, this experiment does make the point it is probably normal for cells to set their basal respiratory rates below their maximal respiratory capacities. This would provide a respiratory cushion that could be utilized in the event of acute energy crisis. This respiratory reserve could provide a valuable target for mitochondrial medicine, and developing pharmacologic approaches that tap this respiratory reserve could prove useful for the treatment of neurodegenerative mitochondriopathies. 

### 3.3. Opportunities for combination therapy

Optimal neurodegenerative mitochondriopathy therapies will slow or prevent progression by interfering with underlying molecular pathologies and also provide symptomatic benefits. As mitochondrial medicine approaches advance, opportunities for using these approaches with treatments designed to influence other pathologies or specific disease symptoms will arise. For example, if dimebon does benefit AD patients and does so via mitochondrial effects, it will be important to test whether dimebon in conjunction with a cholinesterase inhibitor is more efficacious than using these agents separately.

## 4. Conclusions

Mitochondrial perturbations are protean in the neurodegenerative diseases. It is reasonable to consider many of these disorders as neurodegenerative mitochondriopathies. The search for effective therapies should include treatments that target mitochondria or pathways affected by mitochondrial function. In pursuing this, whether mitochondrial dysfunction in a particular disease is likely to have an initiating, intermediate, or downstream role in neurodegenerative cascades requires consideration. It is also necessary to take into account whether a pharmacologic intervention is targeting upstream or downstream mitochondrial pathology. Failure to do so could produce therapies that are ineffective or, if they reduce or abolish compensatory physiology, are even harmful. 

Mitochondrial medicine thus far has failed to revolutionize the treatment of neurodegenerative diseases, but a better understanding of basic mitochondrial physiology and the role mitochondria actually play in different neurodegenerative diseases will guide more sophisticated drug development efforts. It is worth noting two non-pharmacologic interventions that benefit human health, DR and exercise, increase mitochondrial respiration. The mechanisms that mediate the effects of DR and exercise could provide a blueprint for developing agents that enhance cell respiration. It will be interesting to see whether pharmacologic strategies designed to specifically and powerfully enhance neuron respiration can meaningfully benefit the many individuals that already have or are destined to develop one of these devastating diseases. 
